# Supplementary material for: A Novel Spinel Ferrite-Hexagonal Ferrite Composite for Enhanced Magneto-Electric Coupling in a Bilayer with PZT
Source: Sensors (Basel). 2023 Dec 14;23(24):9815. doi: 10.3390/s23249815 (PMC10748018; doi:10.3390/s23249815)
Supplement: Supplementary file 1 [file sensors-23-09815-s001.zip › sensors-2695649-supplementary.pdf]

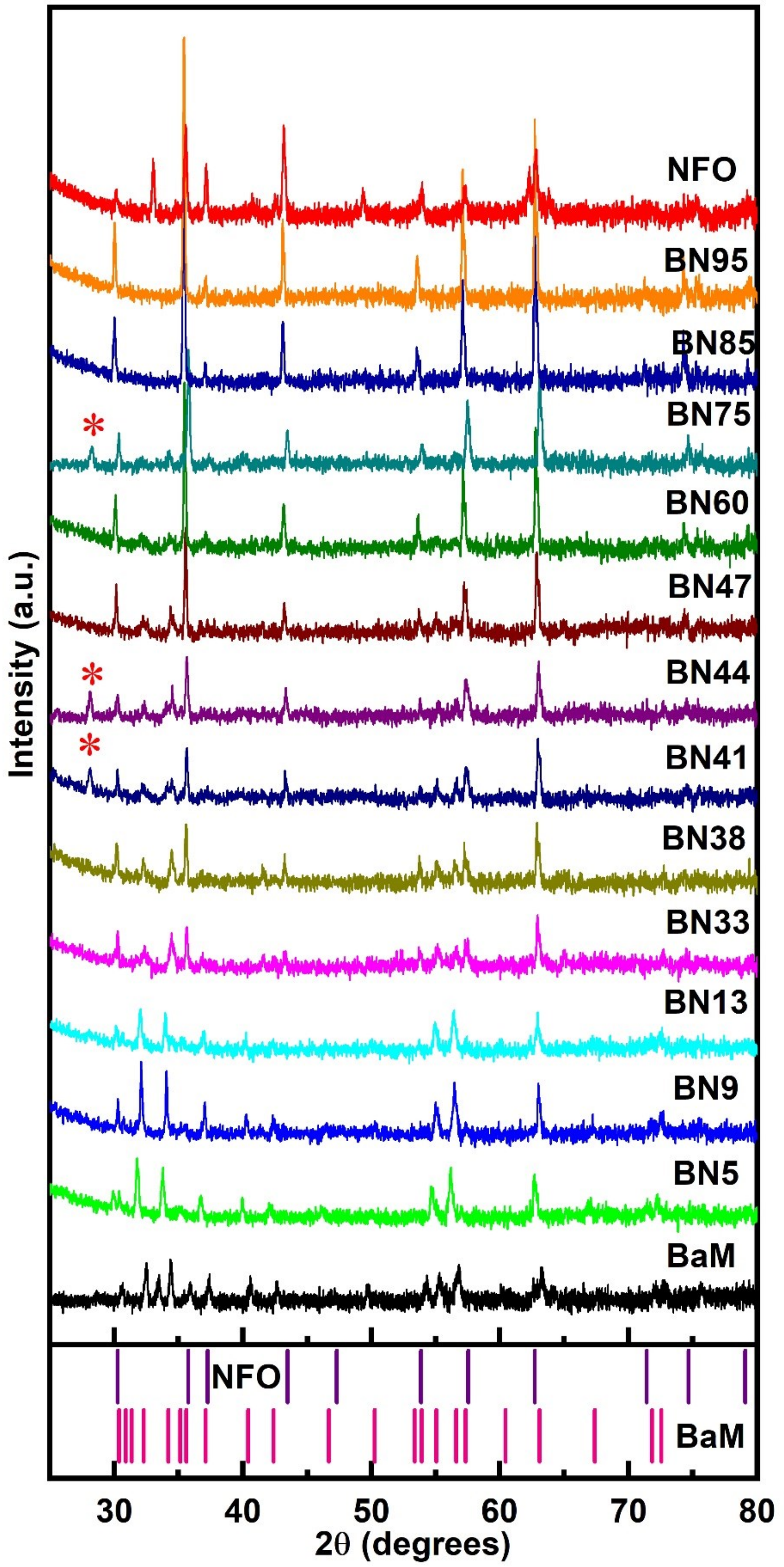

Figure S1. X-ray diffraction patterns of BNx composites. All composites bear the signatures of NFO and BaM. We have plotted the stick patterns for NFO (PDF No. 00-003-0875) and BaM (PDF No. 00-007-0276) in the bottom pane to visualise the one-to-one correspondence of the Braggs' positions of each phase to the respective NFO and BaM lines.

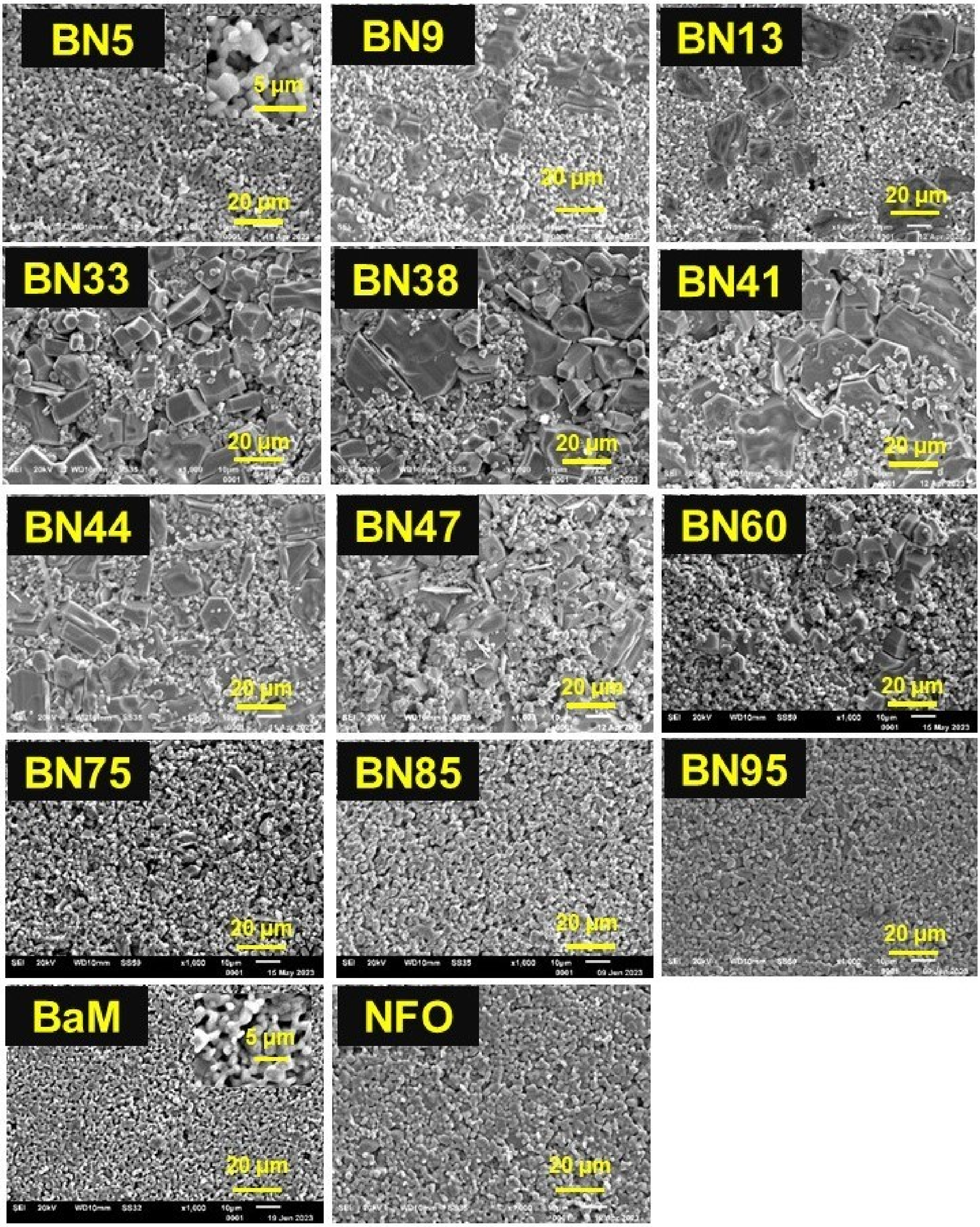

Figure S2. SEM images of BNx (x=5, 9, 13, 33, 38, 41, 44, 47, 60, 75, 85 and 95). Hexagonal BaM grains develop with increasing grain size as the NFO content increases. After BN41 the BaM grains deteriorate in size. SEM images of pure BaM and NFO are also shown at the bottom.

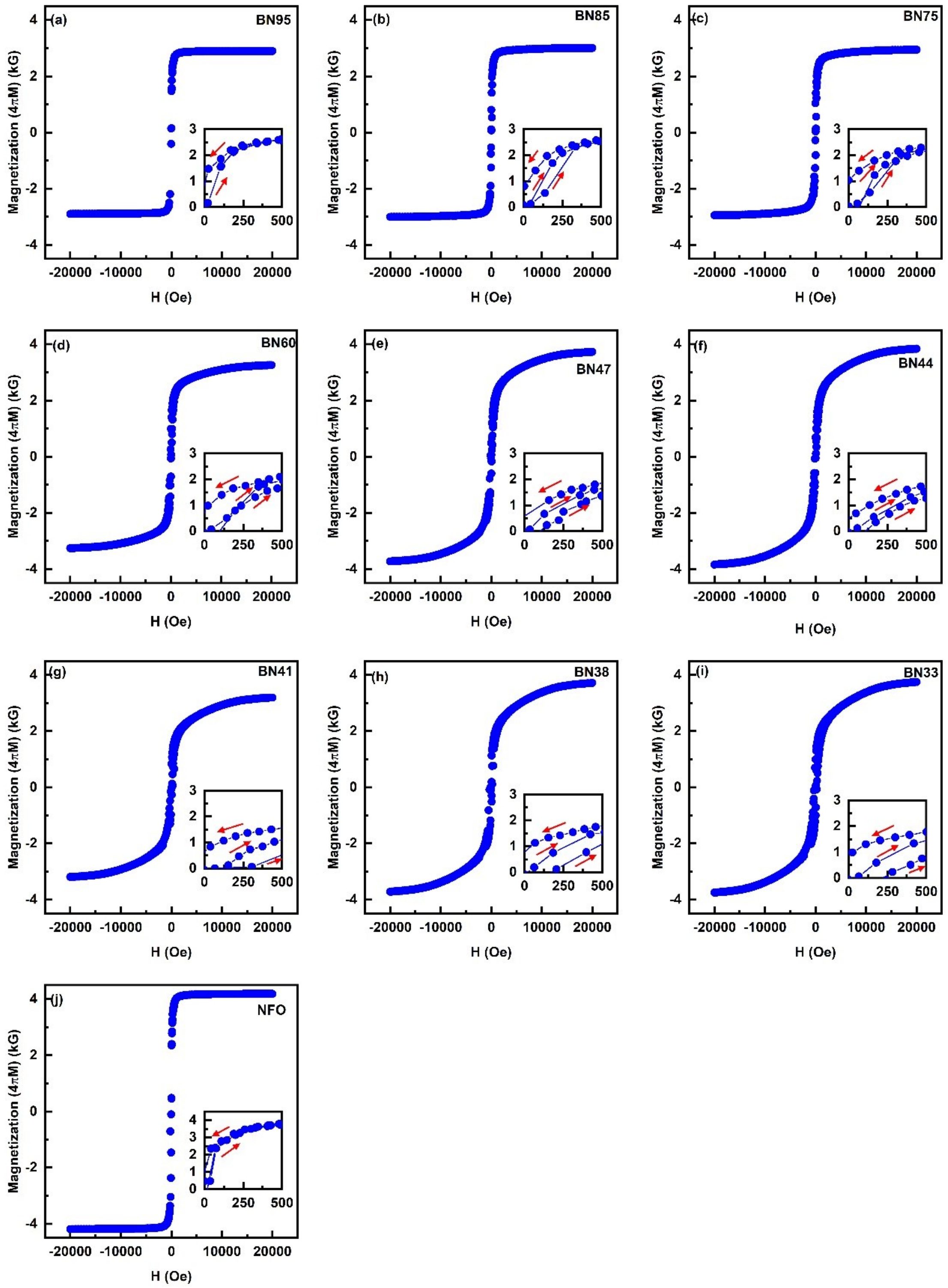

Figure S3. Magnetization vs. magnetic field data for BN composites.

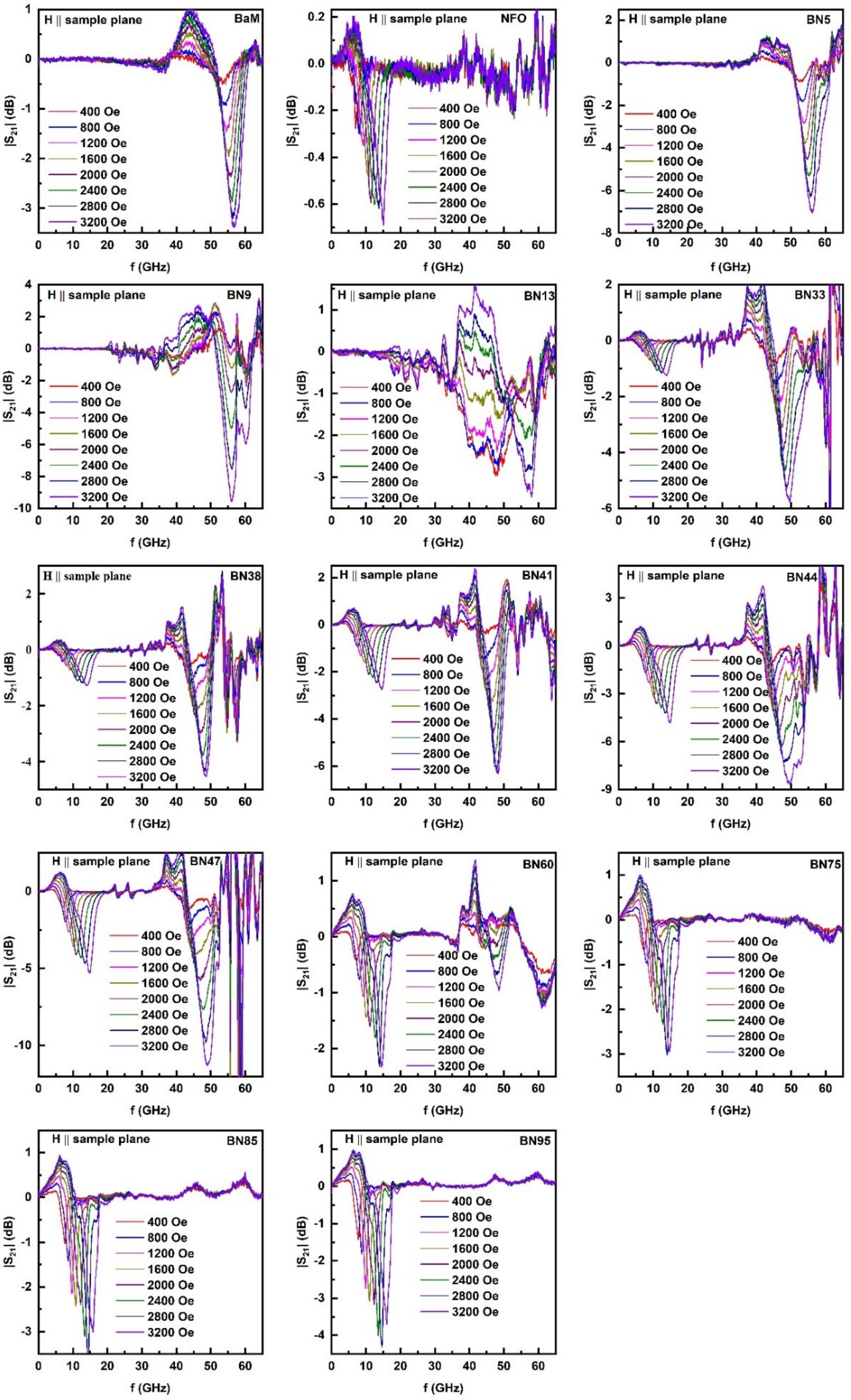

Figure S4. S_{21} vs f profiles showing FMR and magneto-dielectric modes in BNx composites at selected bias magnetic fields.

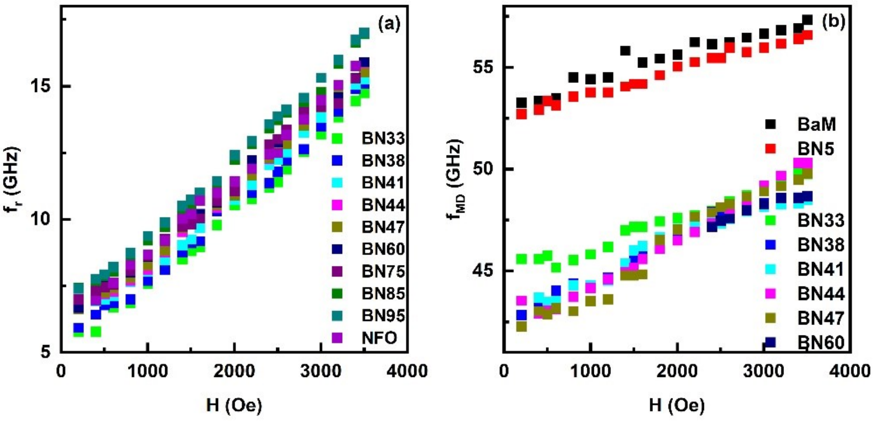

Figure S5. Ferromagnetic resonance frequency of (a) NFO part and (b) magneto-dielectric mode frequencies of BNx composites are plotted as function of external magnetic field (H).

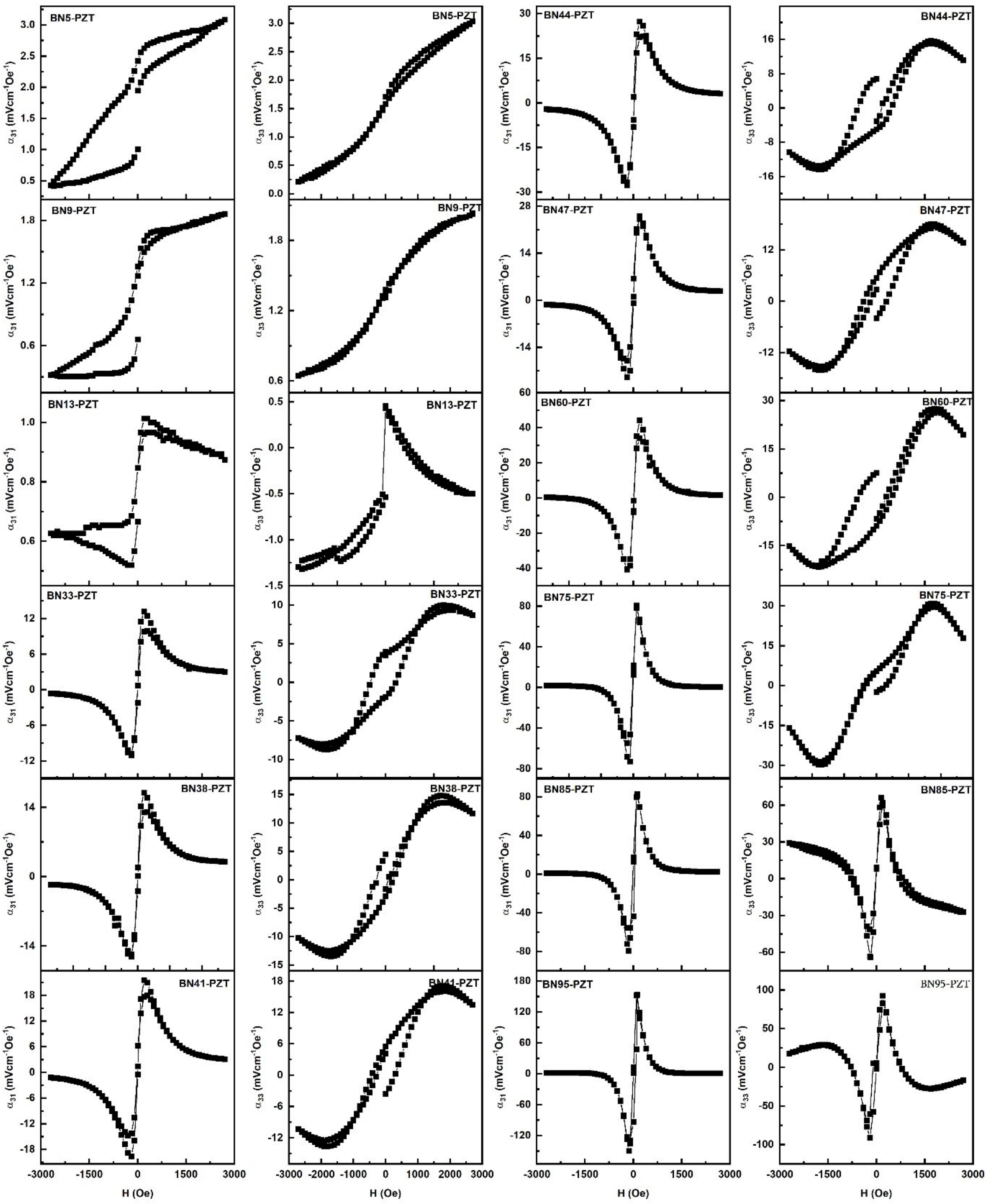

Figure S6. MEVC for BNx-PZT bilayers for in-plane magnetic fields (left) and out-of-plane magnetic fields (right).

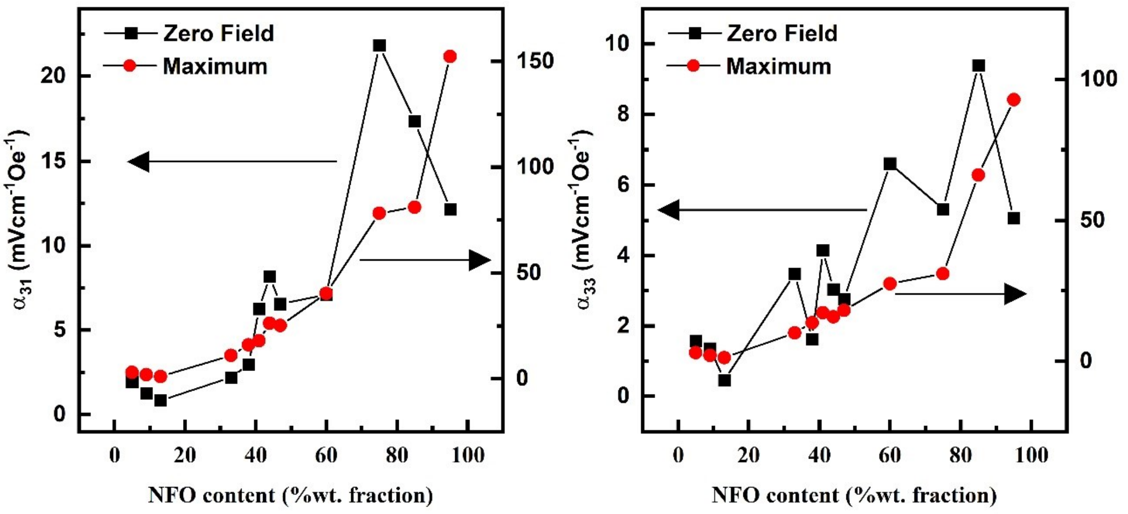

Figure S7. Zero bias and maximum achievable ME coefficient for BNx-PZT bilayers in transverse and longitudinal modes.
